# Psychosocial factors in patients who miss hemodialysis sessions: a single-center retrospective review

**DOI:** 10.1080/0886022X.2026.2694792

**Published:** 2026-07-06

**Authors:** Jessica Dean, Asher Ghafoor, Han Sean Lee, Rajkumar Chinnadurai, Dimitrios Poulikakos

**Affiliations:** a Department of Clinical Health Psychology, Salford Care Organisation, Northern Care Alliance NHS Foundation Trust, Salford, UK; b School of Medical Sciences, University of Manchester, Manchester, UK; c Renal Department, Salford Care Organisation, Northern Care Alliance NHS Foundation Trust, Salford, UK; d Faculty of Biology, Medicine and Health, University of Manchester, Manchester, UK

**Keywords:** Non attendance, hemodialysis, adverse childhood experiences, anxiety, depression

## Abstract

**Background:**

Nonattendance at scheduled hemodialysis (HD) sessions is a frequent form of treatment nonadherence. Psychosocial factors are known contributors, yet the psychological profiles of frequent non-attenders remain underexplored. We conducted a retrospective analysis of patients with recurrent HD nonattendance, defined as missing more than four dialysis sessions during the two-year study period, who were referred to renal psychology services.

**Methods:**

Data were extracted from psychological case notes using a structured pro forma designed by the renal psychology team, and included reason for referral, preexisting mental health issues and history of adverse childhood experiences (ACEs). Information recorded by the dialysis nurses on the electronic patient record on the day of nonattendance for all patients referred to psychology was retrieved and analyzed to identify and categorize recurring themes. Descriptive analysis was conducted for both datasets.

**Results:**

Of 464 patients in the original cohort, 54 met criteria for frequent nonattendance. Twenty-two (40.7%) were referred to psychology, of whom 17 engaged with at least one session. Fifteen out of 17 (88%) of these patients had preexisting mental health conditions, primarily depression (*n* = 12, 71%) and anxiety (*n* = 8, 47%). Confirmed or possible adverse childhood experiences were identified in 88% of assessed patients. Nursing records cited concurrent illness, limited disease understanding, family obligations, and logistical barriers as common reasons for missed sessions.

**Conclusions:**

This study underscores the need for early, integrated psychological assessment within the dialysis care pathway. A trauma-informed, multidisciplinary model may improve access to support and sustain adherence over time and should be prospectively investigated.

## KEY MESSAGES

**What is known:**

Psychosocial factors contribute to hemodialysis nonattendance, but the psychological characteristics of frequent non-attenders and the most effective approaches to improving adherence remain poorly understood.

**What this study adds:**

Among patients with recurrent hemodialysis nonattendance who engaged with renal psychology services, preexisting mental health conditions and confirmed or suspected adverse childhood experiences (ACEs) were highly prevalent, highlighting the potential importance of trauma-informed care model.

**Potential impact:**

These findings support integrating earlier, trauma-informed multidisciplinary care into hemodialysis pathways and highlight the need for prospective studies evaluating whether such approaches improve treatment adherence and patient outcomes.

## Introduction

Nonattendance at scheduled hemodialysis (HD) treatments is a common form of nonadherence that has been consistently linked to poor clinical outcomes, including increased morbidity and mortality [1]. Beyond clinical implications, missed HD sessions disrupt continuity of care and place additional strain on dialysis and hospital services. While logistical factors such as transport issues or intercurrent illness may contribute, a growing body of evidence highlights the critical role of socioeconomic and psychosocial influences on adherence in this population [2].

Adverse childhood experiences (ACEs) have been linked to an increased risk of chronic kidney disease (CKD) [3–5]. While this relationship is partly explained by a higher likelihood of maladaptive coping strategies and engagement in high-risk health behaviors that contribute to obesity, hypertension, and diabetes, it may also reflect the long-term physiological impact of chronic stress. The allostatic load model [6] provides a useful framework for understanding how repeated or sustained exposure to early life stress can lead to dysregulation across neuroendocrine, immune, and metabolic systems, thereby increasing vulnerability to cardio-renal multimorbidity [7]. Early exposure to such adversities can place children at greater risk of serious health challenges later in life, substantially raising their likelihood of developing CKD. ACEs have also been associated with poorer treatment adherence to medications [8–10] and worse health outcomes in adulthood [11], as well as reduced trust in healthcare systems and professionals [12]. Notably, poor medication adherence has been linked to a higher risk of CKD and the onset of end stage renal disease [13]. However, research directly exploring the relationship between ACEs and adherence to dialysis treatment remains limited.

We recently examined dialysis nonattendance in a cohort of 464 maintenance HD patients at a single UK center over a two-year period, reporting both patient characteristics and associated clinical outcomes [14]. Building on that work, the present study focuses on a subset of patients from this cohort who missed multiple dialysis sessions and were referred to renal psychology services.

The aims of this study were to provide a descriptive account of the prevalence and nature of adverse childhood experiences and psychological difficulties in this group, and to explore the acceptability and feasibility of traditional outpatient renal psychology intervention, including dialysis attendance patterns following engagement with the service.

## Methods

We performed a retrospective analysis of adult patients receiving maintenance hemodialysis who demonstrated recurrent nonattendance, defined as missing more than four dialysis sessions between December 2020 and November 2022. Amongst these patients, those referred to the renal psychology service were included in the psychology cohort analysis. The threshold of four or more missed dialysis sessions was selected to identify clinically significant non-adherence, as repeated absences beyond occasional unavoidable events are more likely to reflect persistent behavioral or structural barriers to treatment attendance.

Data on dialysis nonattendance were extracted from incident reports (DATIX) recorded between December 2020 and November 2022. Episodes of nonattendance attributable to concurrent hospitalization were excluded. Patients with severe mental health disorders managed exclusively by external mental health services, including schizophrenia and active substance misuse disorders, were not eligible for referral to the renal psychology service and were, therefore, excluded from the psychology cohort.

**Figure 1.**
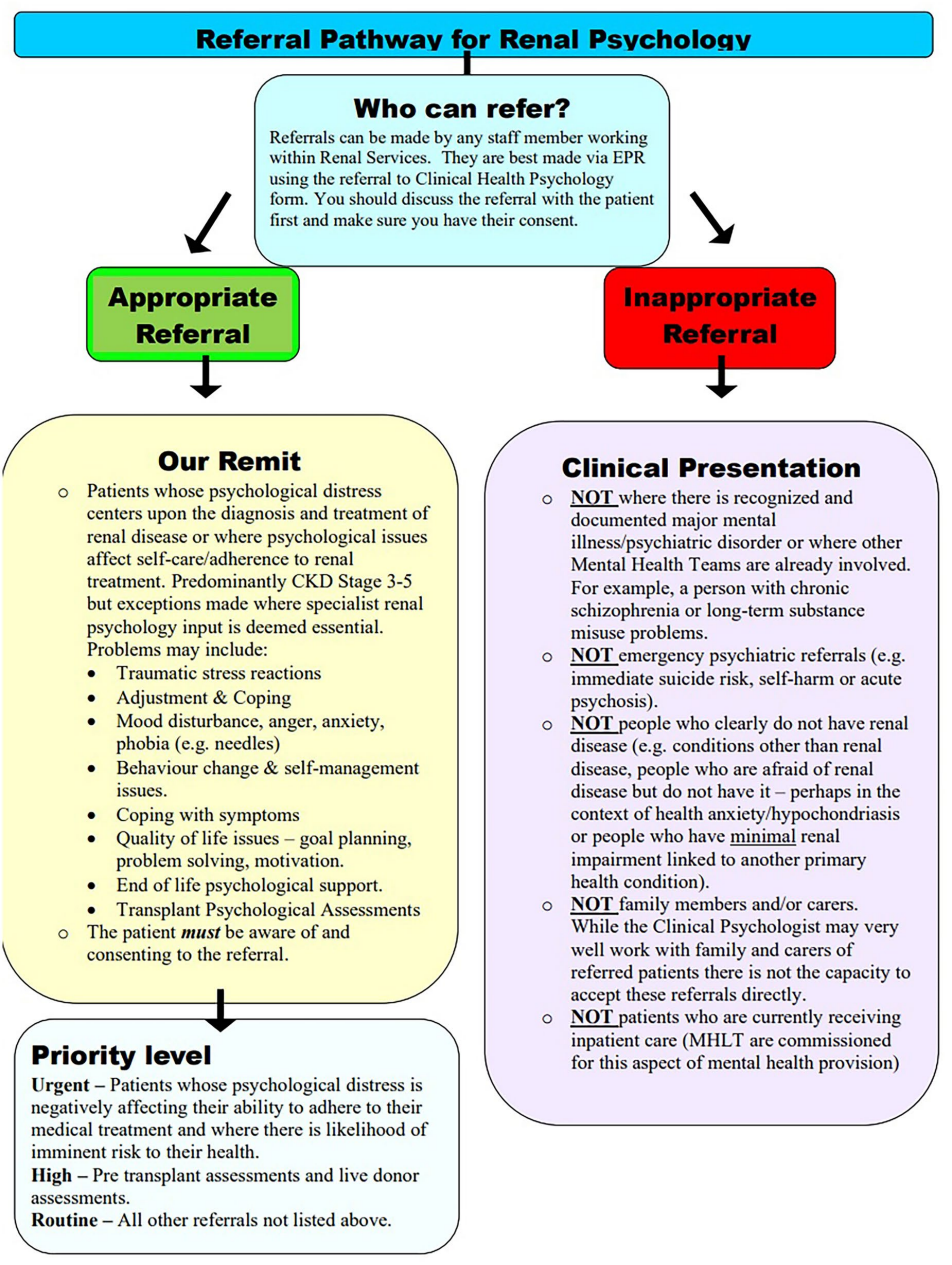
Referral pathway for renal psychology.

Referrals to the psychology service were made according to criteria outlined in Figure 1. Referral decisions are made clinically by the multidisciplinary renal team and were influenced by several factors, including patient willingness to engage and perceived psychological need.

The renal psychology service operates a structured prioritization system for referrals. Urgent referrals include patients whose psychological distress is impacting adherence to treatment and in whom there is concern regarding imminent risk to health, are typically offered an assessment within 2 weeks of referrals. High priority referrals include pre-transplant and live donor assessments while all other referrals are managed as routine, with variable waiting times depending on service demand.

The service utilizes several approaches, including Trauma Informed Cognitive Behavioral Therapy, Acceptance and Commitment Therapy, Compassion Focused Therapy, Eye Movement Desensitization and Reprocessing (EMDR), Solution Focused Therapy, and Motivational Interviewing. Standard outpatient psychology appointments were typically 60 min in duration, with longer 90-min sessions used for more complex interventions such as EMDR. The number of psychological sessions varies substantially depending on clinical complexity, patient engagement, therapeutic goals, and patient preference.

Demographic and clinical variables were obtained retrospectively from the electronic patient record, while psychological information was extracted from renal psychology case notes using a structured pro forma developed by the psychology team (Appendix, Figure A1), and included reason for referral, preexisting mental health issues and history of adverse childhood experiences (ACEs) based on qualitative clinical judgment. In addition, the information recorded by the dialysis nurses on the electronic patient record on the day of nonattendance (Appendix, Figure A2) for all patients referred to psychology was retrieved and analyzed to identify and categorize recurring themes using an inductive approach by three members of the team (AG, JD, DP).

The Index of Multiple Deprivation (IMD), which is a measure of relative deprivation based on geographic areas within the UK, was derived using patients’ postcodes *via* the English Indices of Deprivation 2019 online tool (https://imd-by-postcode.opendatacommunities.org/imd/2019). Adjacent decile scores were then combined to form quintiles from most deprived to least deprived.

These members of the study team independently reviewed and coded records. Formal inter-rater reliability statistics were not calculated due to the exploratory nature and small sample size. Discrepancies were resolved through discussion and consensus, with regular meetings to refine and agree on the thematic framework.

Adverse childhood experiences (ACEs) were classified as ‘confirmed’ when the clinical record contained clear, specific documentation indicating the presence of a defined exposure, such as a formally diagnosed parental mental health condition alongside a description of its impact on the individual’s early life.

In contrast, ACEs were considered ‘suspected’ when notes included suggestive but nonspecific descriptions of adverse experiences (e.g. references to a caregiver’s difficult or distressing behavior) without sufficient detail to establish the presence of a defined ACE. In such cases, the available information indicated a possible exposure, but did not meet the threshold for confirmation.

A comparison was made between the number of missed hemodialysis sessions in the six months before and the six months after patients engaged with the psychology service. Descriptive statistics were used to summarize findings.

This service evaluation using fully anonymized patient data was registered with the Research and Innovation Department of the Northernn Care Alliance NHS Foundation Trust (ID: 24HIP35), and the requirement for individual patient consent was waived.

## Results

Out of the 464 patients in the original cohort, 65 (14%) missed one session, 14 (3%) missed two sessions,16 (3.5%) missed three sessions, and 54 (11.6%) missed four or more sessions. During the period of the study, a total of 81 patients were referred to renal psychology services, with the main reasons being anxiety (22 patients, 27%), depression (38 patients, 47%), and adherence issues (10 patients, 12.4%).

Of the 54 patients who had missed at least 4 HD sessions, 22 patients (40.7%) were referred to psychology. In six cases, medical non-adherence was identified as a primary or secondary reason for referral. Seventeen patients attended at least one appointment, whereas five patients did not attend the initial psychological assessment, or any subsequent appointments and did not respond to follow-up communication; two of these patients had been referred for medical non- adherence. These patients were subsequently discharged without being seen. The reasons for non-engagement are unclear, as detailed case note review was not undertaken.

The mean waiting time for the six patients referred where non-adherence was listed as a primary or secondary reason for referral was 1.8 weeks, compared with 5.8 weeks for the remaining 16 patients referred for reasons other than non-non-adherence.

A majority (92.6%) were in the lower quintiles of the Index of Multiple Deprivation (IMD) (quintiles 1, 2, and 3). The demographic and clinical characteristics of these patients are presented in Table 1.

**Table 1.** Demographics of patients who missed 4 or more dialysis sessions divided into groups based on their engagement with psychology.

Characteristics	Total *N* = 54	Group 1 Not referred to psychology *N* = 32(59.3%)	Group 2 Referred to psychology and attended at least one appointment *N* = 17 (31.4%)	Group 3 Referred to psychology and did not attend any appointment *N* = 5 (9.2%)
Age, years	54 (36–63)	54 (36–66)	56 (43–60)	40 (20–45)
Sex, male	34 (63)	21 (65.6)	11 (64.7)	2 (40)
Ethnicity, White	47 (87)	30 (93.8)	13 (76.5)	4 (80)
Lower IMD Quintiles (1,2,3)	50 (92.6)	29 (90.6)	16 (94.1)	5 (100)
Smoking history	30 (55.6)	19 (59.4)	8 (47.1)	3 (60)
Alcohol history	23 (42.6)	16 (50)	5 (29.4)	2 (40)
Mental health illness	21 (38.9)	12 (37.5)	7 (41.2)	2 (40)
Marital status, single	15 (27.8)	9 (28.1)	4 (23.5)	2 (40)
Diabetes mellitus	21 (39)	13 (40.6)	6 (35.3)	2 (40)
Hypertension	35 (64.8)	20 (62.5)	13 (65.5)	2 (40)
Cardiovascular disease	20 (37)	11 (34.4)	7 (41.2)	2 (40)

Continuous variable (age) is expressed as median (interquartile range). All other categorical variables are expressed as numbers (percentage). IMD: Indices of multiple deprivation (IMD) is a metric of relative deprivation for small, geographic areas of the UK. IMD categorizes these areas into five quintiles based on relative disadvantage, with quintile 1 being the most deprived and quintile 5 being the least deprived.

In the 268 nursing entries related to missed dialysis appointments of the 22 patients referred to psychology, the most common reported reason for dialysis nonattendance was concurrent illness followed by lack of understanding of disease, family commitments and logistics related to the dialysis center (Table 2).

**Table 2.** Thematic presentation of reasons for dialysis nonattendance in the 22 patients who were referred to psychology and had missed more than 4 dialysis sessions.

Reason	Examples of reasons in this category.	Number of times reason was cited for missing dialysis. (268 missed sessions)
Illness	Ill/unwell. admitted to hospital, Emergency Department visit.	69 (25.7%)
No response given	No reason	46 (17.1%)
Lack of understanding of disease	Felt fine, refused, felt thrice weekly “too much”, didn’t feel like it.	38 (14.1%)
Family commitment	Birthdays, weddings, child unwell, family issues,	25 (9.3%)
Dialysis center issues	No side room, waiting times, problem with line, wishing to reschedule.	24 (8.9%)
Busy	Going out, too busy, going away	24 (8.9%)
Treatment Burden	Tired, need rest, want a break	13 (4.8%)
Other	Personal reasons (detail not disclosed). Overslept, Any other issues.	10 (3.7%)
Appointment	Other medical appointment, work meeting, other non-medical appointment.	9 (3.3%)
Transport issues	No transport	6 (2.2%)
Psychological difficulty	Upset, Angry, Depressed.	4 (1.4%)

The number of psychological sessions attended varied widely (Table 3). Decisions to end therapy were made for several reasons, including successful completion of treatment, patient choice, disengagement from the service, repeated nonattendance at appointments, or transfer to alternative services.

**Table 3.** Psychology data and missed dialysis sessions for patients who attended at least one psychology appointment.

	Reason for referral to psychology	Reason for referral to psychology (additional)	Preexisting mental health issue?	Type of previous mental health issue	Previous mental health issue (additional)	ACES	Abuse	Neglect	Substance Misuse	Mental Illness	Divorce/Separation	Domestic Violence	Incarceration	Total number of ACES identified	Psychology Appts Attended	Psychology Appts DNA or Canceled	Number of dialysis sessions missed 6 months prior to psychology input.	Number of dialysis sessions missed 6 months after psychology input.
1	Depression	Medical non-adherence	YES	Substance Dependency	Grief	YES				√		√		2	10	4	8	0
2	Depression	PTSD	YES	PTSD	Depression	NO								0	1	0	0	2
3	Depression	Medical non-adherence	Unclear			Unclear								0	7	3	17	4
4	Anxiety		NO			NO								0	6	6	0	5
5	Anxiety		YES	Addiction		YES		√	√	√	√			4	5	7	14	0
6	Anxiety	Depression	YES	Depression		Unclear								0	8	1	19	22
7	Depression		YES	Depression	Anger	YES	√		√	√				3	8	3	4	2
8	Substance Dependency	Medical non-adherence	YES	Substance Dependency	Depression	YES		√		√	√			3	4	2	1	7
9	Anxiety	Substance Dependency	YES	Substance Dependency	Depression	YES	√			√		√		3	6	1	21	20
10	Anger		YES	PTSD		YES	√		√	√				3	5	4	0	1
11	Anxiety	Depression	YES	Depression		Unclear								0	2	6	0	4
12	Medical non-adherence	Anxiety	YES	Anxiety		YES	√			√	√			3	8	12	10	8
13	Anxiety	Depression	YES	Depression		YES				√	√			2	3	1	0	1
14	Eating Disorder	Anxiety	YES	Eating Disorder		YES	√		√	√	√	√		5	39	8	10	18
15	Depression		YES	Depression		YES	√		√	√	√		√	5	5	5	3	4
16	Depression		YES	Depression		Unclear								0	1	0	1	2
17	Depression		YES	Depression		YES	√			√	√			3	2	1	0	6

ACE: Adverse Childhood Experiences; Appts: appointments; DNA: did not attend; PTSD: Post-Traumatic Stress Disorder.

Among those assessed by the psychology team, 15 of 17 patients (88%) had a documented history of preexisting mental health issues, including depression (*n* = 12, 71%) and anxiety (*n* = 8, 47%). Adverse Childhood Experiences (ACEs) were common, with confirmed ACEs identified in 11 patients (65%) and possible ACEs in an additional 4 patients (24%).

Following psychological engagement, dialysis attendance improved in 6 out of 17 (35.2%) patients who attended at least one psychology session. Improvement was observed in 3 of the 4 patients where medical non-adherence was primary or secondary reason of referral. Psychology data and missed dialysis sessions for patients who attended at least one psychology appointment are presented in Table 3.

## Discussion

Our findings highlight the high prevalence of preexisting mental health difficulties among patients who miss dialysis sessions in line with existing literature [15,16]. To our knowledge this is the first report of high prevalence of ACEs in maintenance HD patients under the care of renal psychology services. The observed low referral and engagement rates among frequent non-attenders highlight the complex psychological, social, and practical barriers faced by this vulnerable patient group, underscoring the need for a proactive multidisciplinary approach to improve awareness, identify unmet psychological needs, and consider targeted interventions.

In addition to childhood adversities contributing to the development of CKD [3–5], patients with end-stage renal disease may encounter medical trauma related to diagnosis, invasive procedures, or ongoing dialysis [17], which can lead to distress, avoidance, and poor mental health. This experience may further be intensified by witnessing the suffering of other patients [18]. Several mechanisms may link ACEs to an increased risk of non-adherence [19] and dialysis nonattendance. The emotional impact of ACEs can impair self-protective capacities, predisposing individuals to what has been described as a ‘trauma-organized’ lifestyle [19], characterized by focus on immediate needs and diminished regard for long-term benefits. Such patterns are increasingly recognized as predictors of non-adherence and engagement in risky health behaviours [20]. Our findings highlight the need for further research into the impact of ACEs on dialysis nonattendance, as well as the potential value of trauma-informed interventions aimed at improving adherence.

Although the study was not designed or sufficiently powered to assess the impact of renal psychological interventions on treatment adherence, dialysis attendance improved in 35.2% of patients during the six months following psychological engagement. This observed modest improvement highlights the complexity of improving and maintaining adherence and the importance of tailored psychological strategies that reinforce behavior over time. Prior studies have shown that the positive effects of adherence interventions diminish or become undetectable by 12 months [21]. Embedding psychological care within the dialysis unit and delivering support through the multidisciplinary team (MDT), rather than relying on conventional outpatient appointments, may enhance both accessibility to psychological care and the sustainability of behavioral change. A trauma-informed approach [22] can provide the appropriate framework to enable a tiered, personalized model of care in which lower intensity educational, cognitive behavioral and counseling interventions can be delivered by the nursing team (supported by specialist renal psychologists through teaching, training and consultation) [23]. Higher intensity, trauma informed, evidence based psychological therapy can then be delivered direct by psychologists where required [24].

Limitations of this report include the small sample size and the retrospective nature of the study. Although the study focused on frequent non-attenders referred to psychology services, only 4 of the 17 frequent non-attenders who were reviewed by the psychology team had non-adherence to medical treatment explicitly recorded as the reason for referral. Moreover, not all patients with recurrent nonattendance were referred to psychology, and psychological assessment data were completed by only 17 of the 54 identified frequent non-attenders, which may limit the generalizability of the findings to the wider population of non-adherent dialysis patients. In addition, the sample excluded patients with major mental health disorders – such as schizophrenia or substance misuse – who are managed by other Mental Health Teams and therefore cannot be referred to the renal psychology service. Patients experiencing substance misuse, which is linked to ACEs [25], frequently have difficulty adhering to the complex requirements of dialysis, often due to unstable living situations and competing demands such as obtaining drugs and financial resources [26].

Additionally, the retrospective exploratory design, lack of standardized pre- and post-intervention assessments, and absence of an adherent control group limited our ability to determine whether the observed psychological characteristics were specific to non-adherent patients or representative of the wider dialysis population. Furthermore, the reasons for discontinuation of psychological therapy were not systematically captured within the data collection proforma and, combined with the small sample size, this limited further analysis of the variability in the number of psychological sessions provided.

Furthermore, the thematic analysis was challenging because some single responses recorded in the electronic patient records could be interpreted as fitting multiple themes, resulting in overlap—particularly between ‘treatment burden’ and ‘lack of understanding of disease’. This difficulty largely stemmed from nonstandardized data collection and the absence of follow-up questions that might have clarified which theme a response belonged to. For this reason, illustrative examples for each category are provided in Table 2.

In conclusion, our study underscores the need for further research into the prevalence and effects of ACEs in hemodialysis patients as well as for multicenter research trials that can inform pathways to proactively identify and support individuals who struggle to adhere to their dialysis treatment. Earlier intervention, ideally integrated within the dialysis care pathway, may help prevent repeated nonattendance and its associated clinical risks.

Future research should prospectively evaluate trauma-informed, tiered psychological care models within hemodialysis services, combining staff training to deliver low-intensity interventions with specialist renal psychology support for patients with more complex needs. Establishing clear criteria for escalation and defining appropriate intervention types for individuals with complex trauma histories will be essential. Universal screening using validated tools such as the PHQ-9 [27] and GAD-7 [28] could provide a structured approach to assessing depression and anxiety, while structured ACE questionnaires could standardize the identification of adverse childhood experiences. Integrating these measures with clinical judgment may improve the consistency and accuracy of psychological assessment, help identify patients most likely to benefit from tiered support, and facilitate evaluation of trauma-informed interventions. These approaches could be tested in a multicenter cluster randomized or stepped-wedge trial, assessing outcomes such as dialysis attendance, patient engagement, psychological wellbeing, and healthcare utilization, alongside a process evaluation to examine feasibility, acceptability, and fidelity of implementation.

## Supplementary Material

Appendix.pdf

## Data Availability

The authors confirm that the data supporting the findings of this study are available within the article.

## References

[1] Saran R, Bragg-Gresham JL, Rayner HC, et al. Nonadherence in hemodialysis: associations with mortality, hospitalization, and practice patterns in the DOPPS. Kidney Int. 2003;64(1):254–262. doi: 10.1046/j.1523-1755.2003.00064.x. 12787417

[2] Clark S, Farrington K, Chilcot J. Nonadherence in dialysis patients: prevalence, measurement, outcome, and psychological determinants. Semin Dial. 2014;27(1):42–49. doi: 10.1111/sdi.12159. 24164416

[3] Shen Z, Li C, Fang Y, et al. Association between adverse childhood experiences with chronic kidney diseases in middle-aged and older adults in mainland China. Sci Rep. 2025;15(1):6469. doi: 10.1038/s41598-025-91232-4. 39987259 PMC11847003

[4] Clark K, Al-Uzri A. The prevalence of adverse childhood experiences in adults with CKD: PUB310. J Am Soc Nephrol. 2021;32(10S):851–851. doi: 10.1681/ASN.20213210S1851d.

[5] Zhang K, Wang Y, Sun Y, et al. Self-reported childhood adversity, unhealthy lifestyle and risk of new-onset chronic kidney disease in later life: a prospective cohort study. Soc Sci Med. 2024;341:116510. doi: 10.1016/j.socscimed.2023.116510. 38159486

[6] Finlay S, Roth C, Zimsen T, et al. Adverse childhood experiences and allostatic load: a systematic review. Neurosci Biobehav Rev. 2022;136:104605. doi: 10.1016/j.neubiorev.2022.104605. 35278597

[7] Zhu Q, Xu L, Fan Z, et al. Allostatic load and progression of cardio-renal multimorbidity: a UK biobank study. PLoS One. 2026;21(1):e0339576. doi: 10.1371/journal.pone.0339576. 41490086 PMC12768364

[8] Whetten K, Shirey K, Pence BW, CHAT Research Team., et al. Trauma history and depression predict incomplete adherence to antiretroviral therapies in a low income country. PLoS One. 2013;8(10):e74771. PMID: 24124455; doi: 10.1371/journal.pone.0074771. 24124455 PMC3790775

[9] Korhonen MJ, Halonen JI, Brookhart MA, et al. Childhood adversity as a predictor of non-adherence to statin therapy in adulthood. PLoS One. 2015;10(5):e0127638. doi: 10.1371/journal.pone.0127638. 26011609 PMC4444303

[10] Kriegbaum M, Kildemoes HW, Rasmussen JN, et al. Childhood socioeconomic position, young adult intelligence and fillings of prescribed medicine for prevention of cardiovascular disease in middle-aged men. BMJ Open. 2014;4(1):e004178. doi: 10.1136/bmjopen-2013-004178. PMC3902485 24441056

[11] Hughes K, Bellis MA, Hardcastle KA, et al. The effect of multiple adverse childhood experiences on health: a systematic review and meta-analysis. Lancet Public Health. 2017;2(8):e356–e366. doi: 10.1016/S2468-2667(17)30118-4. 29253477

[12] Bellis MA, Hughes K, Ford K, et al. Associations between adverse childhood experiences and trust in health and other information from public services, professionals and wider sources: national cross sectional survey. BMJ Public Health. 2024;2(1):e000868. doi: 10.1136/bmjph-2023-000868. 40018105 PMC11812900

[13] Cedillo-Couvert EA, Ricardo AC, Chen J, CRIC Study Investigators, et al. Self-reported medication adherence and CKD Progression. Kidney Int Rep. 2018;3(3):645–651. doi: 10.1016/j.ekir.2018.01.007. 29854972 PMC5976857

[14] Chinnadurai R, Dean J, Rengarajan S, et al. Hemodialysis non-attendance: patient characteristics and outcomes in a Single Renal Center in North West England. Hemodial Int. 2025; 29(3):363–370. doi: 10.1111/hdi.13227. 40051030 PMC12287893

[15] Chan KE, Thadhani RI, Maddux FW. Adherence barriers to chronic dialysis in the United States. J Am Soc Nephrol. 2014;25(11):2642–2648. doi: 10.1681/ASN.2013111160. 24762400 PMC4214530

[16] Weisbord SD, Mor MK, Sevick MA, et al. Associations of depressive symptoms and pain with dialysis adherence, health resource utilization, and mortality in patients receiving chronic hemodialysis. Clin J Am Soc Nephrol. 2014;9(9):1594–1602. doi: 10.2215/CJN.00220114. 25081360 PMC4152801

[17] Hercz G. The trauma of dialysis initiation. J Am Soc Nephrol. 2017;28(10):2835–2837. doi: 10.1681/ASN.2017020212. 28848001 PMC5619974

[18] Eagle G, Kaminer D. Continuous traumatic stress: expanding the lexicon of traumatic stress. Peace Conflict. 2013;19(2):85–99. doi: 10.1037/a0032485.

[19] Oral R, Ramirez M, Coohey C, et al. Adverse childhood experiences and trauma informed care: the future of health care. Pediatr Res. 2016;79(1-2):227–233. doi: 10.1038/pr.2015.197. 26460523

[20] Chanlongbutra A, Singh GK, Mueller CD. Adverse childhood experiences, health-related quality of life, and chronic disease risks in rural areas of the United States. J Environ Public Health. 2018;2018:7151297–7151215. doi: 10.1155/2018/7151297. 30112012 PMC6077617

[21] Murali KM, Mullan J, Roodenrys S, et al. Strategies to improve dietary, fluid, dialysis or medication adherence in patients with end stage kidney disease on dialysis: a systematic review and meta-analysis of randomized intervention trials. PLoS One. 2019;14(1):e0211479. doi: 10.1371/journal.pone.0211479. 30695068 PMC6350978

[22] Breckenridge T, Trauma A. Informed care model: addressing adverse childhood experiences in patients with end-stage kidney disease. J Nephrol Soc Work. 2022;46(1):p16.

[23] Bonnet K, Bergner EM, Ma M, et al. African American Patients’ perspectives on determinants of hemodialysis adherence and use of motivational interviewing to improve hemodialysis adherence. Clin J Am Soc Nephrol. 2025;20(1):88–100. doi: 10.2215/CJN.0000000580. 39412894 PMC11737444

[24] Matteson ML, Russell C. Interventions to improve hemodialysis adherence: a systematic review of randomized-controlled trials. Hemodial Int. 2010;14(4):370–382. doi: 10.1111/j.1542-4758.2010.00462.x. 20796047

[25] Broekhof R, Nordahl HM, Tanum L, et al. Adverse childhood experiences and their association with substance use disorders in adulthood: a general population study (Young-HUNT). Addict Behav Rep. 2023;17:100488. doi: 10.1016/j.abrep.2023.100488. 37077505 PMC10106480

[26] Scott JK, Taylor DM, Dudley CRK. Intravenous drug users who require dialysis: causes of renal failure and outcomes. Clin Kidney J. 2018;11(2):270–274. doi: 10.1093/ckj/sfx090. 29644070 PMC5887625

[27] Levis B, Benedetti A, Thombs BD, DEPRESsion Screening Data (DEPRESSD) Collaboration. Accuracy of Patient Health Questionnaire-9 (PHQ-9) for screening to detect major depression: individual participant data meta-analysis. BMJ. 2019;365:l1476. doi: 10.1136/bmj.l1476. 30967483 PMC6454318

[28] Löwe B, Decker O, Müller S, et al. Validation and standardization of the generalized anxiety disorder screener (GAD-7) in the general population. Med Care. 2008;46(3):266–274. doi: 10.1097/MLR.0b013e318160d093. 18388841

