# Supplementary material for: Psychosocial factors in patients who miss hemodialysis sessions: a single-center retrospective review
Source: Ren Fail. 2026 Jul 6;48(1):2694792. doi: 10.1080/0886022X.2026.2694792 (PMC13348120; doi:10.1080/0886022X.2026.2694792)
Supplement: Appendix.pdf [file IRNF_A_2694792_SM9553.pdf]

Appendix

Figure A1

Psychology Casenote Screening proforma

Reason referred to psychology:	*Primary reason stated in referral form.*
Additional reason referred to psychology:	*Any additional reasons stated.*
Pre-existing mental health issue:*	*YES/NO*
*prior to commencing dialysis	
Type of pre existing mental health issue:	*Documented mental health condition*
Additional pre-existing mental health issue:	*Documented mental health condition(s)*
Evidence of exposure to ACE's when reviewing notes?	*YES/NO/UNCLEAR**

** unclear indicates some evidence of ACE but not enough detail to be sure.*

<u>**Type of ACE documented**</u>		***(tick all present)***
ABUSE (emotional, physical or sexual)	☐	
NEGLECT (physical or emotional)	☐	
HOUSEHOLD SUBSTANCE MISUSE	☐	
PARENTAL MENTAL ILLNESS *inc suicide	☐	
PARENTAL DIVORCE or SEPERATION	☐	
WITNESSING DOMESTIC VIOLENCE	☐	
HOUSEHOLD MEMBER INCARCERATION	☐	

Total Number of ACES identified.	*Insert total number*
Number of Psychology Appointments Attended.	*Insert total number*
Number of Psychology Appointments DNA/Cancelled.	*Insert total number*

ACE: Adverse childhood experiences

Figure A2

Renal Haemodialysis Non-Attendance Structured Proforma

on Electronic Patient Record

- Has the patient contacted the unit and informed staff they will not be attending for haemodialysis: Yes/No

- Based on most recent attendance is the patient known to have capacity: Yes/No

- Has contact been made with the patient: Yes/No

- State why the patient unable to attend and what advice you have given:

  Free text

- Has an alternative slot on the same day been offered: Yes/No

- Has an alternative slot on the same day been accepted: Yes/No

- Date and time of same day appointment:
